# Robotic Retromuscular (Recurrent) Parastomal Hernia Repair (r-Pauli-Repair) With Synthetically Reinforced Biological Mesh; Technique, Early Experience, and Short-Term Follow-Up

**DOI:** 10.3389/jaws.2023.12059

**Published:** 2023-12-13

**Authors:** A. L. A. Bloemendaal

**Affiliations:** Department of Surgery, Reinier de Graaf Gasthuis, Delft, Netherlands

**Keywords:** parastomal hernia repair, robotic, retromuscular, biological mesh, abdominal wall surgery

## Abstract

**Introduction:** Parastomal hernia repair remains a challenge. We describe a robotic retromuscular non-keyhole mesh repair using a synthetically reinforced biological mesh (Ovitex) for the repair of complex and/or recurrent parastomal hernia and technical modifications we made along the way to improve our technique.

**Methods:** All patients underwent the described retromuscular parastomal hernia repair. Data was collected in a database and a retrospective analysis was performed on direct postoperative results and early follow-up.

**Results:** Eleven patients underwent the operation. Median follow-up was 12 months. Median LOS was 6 days. Two recurrences occurred. One patient suffered postoperative hematoma and skin necrosis, which healed completely, but did lead to a recurrence. One patient had a significant seroma, which subsided without intervention. Both recurrences were reoperated, and a local repair was performed.

**Conclusion:** This paper is the first to describe a modified robotic Pauli repair for complex and recurrent parastomal hernia, using a synthetically reinforced biological mesh. Results are satisfying so far, especially considering the complexity of the cases.

## Introduction

Almost 1 million people in the US have an ostomy and 100,000 more are created yearly [1]. European numbers are less clear, but estimated at 700,000 in a position paper by Eucomed in 2012 [2].

Ostomy creation has a high rate of complications, reports varying from 2.9% to >80%, of which parastomal hernia (PSH) is one of the most common, leading to a major medical and personal burden [3]. Furthermore, repairs of PSHs are known to have high recurrence rates [4]. Due to low complication rate and acceptable recurrence rate, the European Hernia Society guideline recommends a so-called modified Sugarbaker operation, in which a synthetic flat mesh is placed intraperitoneally over the stoma conduit [5]. This technique may, at present, be considered the standard of care for parastomal hernia repair, but the direct contact of the mesh with the bowel may cause devastating mesh related complications, like mesh-erosion, mesh-infection, fistulae, and bowel perforation [6–9]. In search of further improvement of treatment of PSH, Pauli et al. described a PSH repair with retromuscular placement of the non-keyhole mesh and a limited unilateral posterior component separation/transversus abdominis release (PCS/TAR) with a recurrence rate of 4.5% at 10 months [10]. The technique was subsequently modified to a robotic repair to combine the benefits of minimally invasive surgery and retromuscular mesh placement [11]. In this technique, however, there will still be intimate contact between the stoma conduit and the synthetic mesh, which may again lead to devastating mesh-related complications, necessitating major revisional and salvage surgery [12].

Although the use of biological meshes has been strongly debated in ventral hernia repair, with a randomized trial showing inferior results for biological mesh [13], an important difference is the necessary intimate contact between mesh and bowel in a retromuscular PSH repair compared to a retromuscular ventral hernia repair in contaminated abdominal wall repair. Miller et al. report on a comparison between the two mesh types in PSH repair, with no superiority for the biological mesh shown [14]. However, this report was a *post hoc* analysis of the abovementioned study (comparing hernia repairs in contaminated ventral hernia repairs), which may lead to a bias when comparing the results for concomitant PSH repair. Furthermore, all patients in this study underwent an open operation.

To date no reports have been published on the use of a biological mesh in a robotic retromuscular parastomal hernia repair. In this article we will describe the development of the technique and our early experience with this method and the modifications we made along the way in the search for the optimal treatment of complex parastomal hernia repair.

## Methods

All patients were operated in the same centre (Reinier de Graaf Gasthuis, Delft, the Netherlands) and by the same surgeon (AB). Oral consent for publication was obtained from all patients.

The operations were performed on a DaVinci Xi robotic system (Intuïtive Surg, Sunnyvale USA). All meshes placed were Ovitex 1S with polypropylene reinforcement [TelaBio, Malvern, PA, USA].

### Patient Selection and Follow-Up

All patients for whom a parastomal hernia repair was considered underwent a CT abdomen in our hospital. Based on clinical findings patients were offered a robotic, laparoscopic or an open approach. In case of clear contraindications for pneumoperitoneum of lengthy operation either an open direct repair, or a laparoscopic Sugarbaker repair was discussed.

The choice of mesh (synthetic or biologic) was discussed in depth with the patient as there is no clear evidence for superiority on one mesh over the other. Patients’ preference was leading.

BMI, smoking, diabetes, immunosuppressants and number of previous operations were not considered to be hard contraindications. Smoking cessation was advised but not demanded.

Postoperative follow-up was standard for all patient: at 2 weeks by phone and at 6 weeks, 3 and 12 months a physical examination. In case of pain or doubt imaging was performed.

### Operative Procedure

Patients received a single dose intrathecal morphine preoperatively and were positioned in supine position under general anaesthesia. Prophylactic cefazoline/metronidazol was administered. A continuous drip of muscle relaxant was administered to achieve deep relaxation throughout the procedure. An indwelling urinary catheter was inserted.

In the first three procedures the peristomal area was kept uncovered for placement of transfascial sutures. These sutures were abandoned as from the fourth case and the stoma area was completely covered in sterile drapes to decrease skin exposure and increase sterility.

Insufflation to 15 mmHg was achieved with a Veress needle to the left upper quadrant (Palmer’s point). Three robotic trocarts were then placed contralateral to the stoma in the mid-axillary line. The robot was docked, and the camera and instruments were inserted. Adhesiolysis was performed as necessary.

The stoma conduit was mobilized as much as possible. In case of recurrent PSH after mesh placement, the mesh was left *in situ*, unless it was easily removed, or removal looked necessary due to contamination. Our goal was to achieve a stoma conduit hanging freely into the abdomen. Retromuscular dissection towards the stoma was started at the midline ipsilateral to the stoma. In case of a concomitant midline hernia a robotic TARUP was performed as described by Muysoms et al. [15] and dissection was started into the retromuscular plane contralateral to the stoma.

**FIGURE 1:**
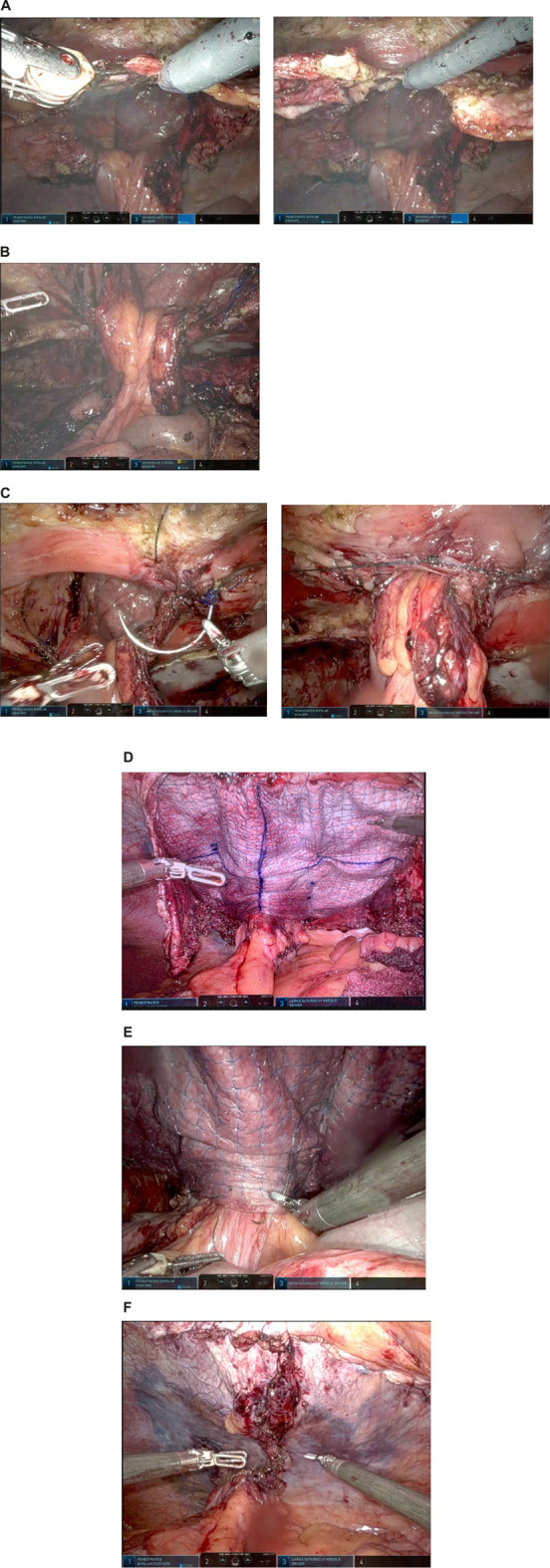
Operative steps of robotic retromuscular parastomal hernia repair with biological mesh. **(A)** Dissection of the posterior layer to facilitate dissection around the conduit. **(B)** Fully dissected and “free hanging” stoma conduit. **(C)** Approximation of the hernia. **(D)** Mesh placement and fixation. **(E)** Suture fixation of conduit to mesh to prevent sliding of the conduit. **(F)** Closure of posterior layer.

In recurrent cases with mesh present, the posterior rectus fascia was dissected toward the stoma to facilitate the dissection around the stoma (Figure 1A). Mesh was mostly present and traction to the posterior layer was helpful in dissecting the layer carefully away from the stoma conduit. Once the conduit was free from the posterior layer a transversus abdominis release was performed to dissect into the preperitoneal layer (or pre-fascia transversalis layer) for further lateral dissection (Figure 1B). The plane was developed as far as possible laterally and then dissected laterally, where lateralization of the conduit was planned.

A 15 mm trocart was placed in the upper abdominal quadrant for introduction of sutures and mesh. If the expected mesh was too large for a 15 mm port, a small Alexis [Applied Medical, Rancho Santa Margarita, USA] was placed with a cap on for placement of larger mesh.

The hernia defect was approximated around the conduit with V-loc 0 [Medtronic, Fridley, USA] (Figure 1C). The midline hernia, if present, was also closed with V-loc 0.

The stoma conduit was lateralized as far as possible by fixing it to the lateral abdominal wall with v-loc 3/0. Measurement for mesh-size was performed.

The Ovitex mesh was cut to size if necessary and was soaked in gentamicin solution [240 mg gentamicin in 400 mL saline) for 4 min. The mesh was introducible through a 15 mm trocar if not larger than 16 × 20 cm.

In the first three cases a 3/0 vicryl suture [Johnson&Johnson, Raritan, USA] had been placed in the corners for transcutaneous suture retrieval and mesh fixation. This technique was found to be time consuming and was replaced by the placement of loosely placed sutures to the corners at the start of fixation. The mesh was placed with the reinforced side towards the abdomen. The side without polypropylene reinforcement was placed towards the stoma conduit to decrease the chance of erosion.

The mesh was fixated with a 3/0 v-loc in the corners after placement. Onwards, the edges of the mesh were fixated with 3/0 v-loc. Then, to decrease dead-space and to prevent creasing of the mesh, it was fixed with further v-loc 3/0 sutures alongside the stoma and in a second “crown” (Figure 1D).

In later cases, to prevent sliding of the conduit behind the mesh, the stoma conduit itself was fixed to the mesh with V-loc 3/0 at the mesh edge almost circumferentially and again fixed to the abdominal wall to decrease the “tunnel” lateral to the conduit, where recurrences are known to occur (Figure 1E).

In case of a concomitant midline hernia -not covered by the Ovitex mesh-a second mesh (Progrip [Medtronic, Fridley, USA]) was cut to size and placed.

The posterior layer was closed with V-loc 2/0 sutures from the stoma towards the midline. The incision of midline posterior layer was also closed with V-loc 2-0 (Figure 1F). In one case the posterior layer closure was not satisfactory at the basis of the conduit. An omental flap was placed over the uncovered mesh.

In one case, after previous synthetic mesh erosion and infection necessitating explantation of mesh and consequent absence of posterior layer with a recurrence of hernia, we performed an intraperitoneal onlay mesh (IPOM) placement in Sugarbaker configuration. In this case the mesh was placed with the reinforced side towards the stoma, as would be normally done in case of IPOM placement with this mesh. Fixation of the mesh was as described above for the retromuscular placement.

No drains were placed in all cases.

The procedure was concluded by closure of fascia at the 15 mm port. The skin was closed with monocryl (Johnson&Johnson, Raritan, USA).

## Results

Eleven patients were operated with above-mentioned technique. A planned 12th case was converted immediately to an open direct repair due to severe adhesions and was left out of further analysis. All but one PSHs were large at >5 cm (EHS type III/IV). Six out of eleven patients had undergone previous PSH repairs with five out eleven patients having synthetic mesh present at the operation. Patient and operative characteristics are shown in Table 1.

**TABLE 1:** Characteristics of operated patients, hernia, previous operations and early results.

Pt	Age	BMI	Smoking	Diabetes	Immune suppressants	ASA	Stoma	EHS	Previous repairs	Mesh present	Mesh layer	Mesh placed (cm)	Complications	CD	LOS	Recurrence	Follow-up (months)
1	70	30	no	no	no	2	EC	I	2	Yes	RM	16 × 20	No	0	7	Yes	19
2	68	33	no	no	no	2	EC	III	1	Yes	IPOM	16 × 20	Seroma, port hernia	1	5	No	18
3	65	22	no	no	no	2	UC	III	0	No		12 × 10	No	0	6	No	15
4	72	36	yes	no	yes	3	UC	III	1	No		12 × 10	No	0	7	No	13
5	69	35	no	no	no	3	EC	III	0	No		18 × 20	Seroma	1	8	No	12
6	77	25	no	no	no	2	EC	III	0	No		16 × 18	No	0	11	No	12
7	68	31	no	no	no	2	EC	IV	2	Yes	IPOM	20 × 25	No	0	6	No	9
8	57	30	yes	no	no	2	EC	IV	4	Yes	IPOM	16 × 20	Hematoma, skin necrosis	2	6	Yes	9
9	69	28	yes	no	no	2	EC	III	2	Yes	RM	16 × 20	No	0	6	No	8
10	66	32	no	no	no	2	EC	IV	0	No		16 × 20	No	0	5	No	7
11	69	34	no	yes	no	3	EC	III	0	No		16 × 20	No	0	7	No	3
Median	69	31													6		12

EC, End colostomy; UC, Urinary conduit; RM, Retromuscular; IPOM, Intraperitoneal onlay mesh; CD, Clavien Dindo classification; LOS, Lenght of stay.

Patients were routinely seen in clinic at 6 weeks, 3 months and 1 year postoperatively (if applicable). Apart from physical examination no standard radiological examination was performed, unless deemed necessary.

### Complications and Reoperations

There were no intraoperative complications.

The first patient developed a bulge after 3 months, which was found to be based on sliding of the colonic stoma conduit behind the mesh, and not on a parastomal herniation of abdominal content. The complaint was mild, with no change of function or troubles in caretaking of the stoma. Patient, however, insisted on a reoperation, in which a direct repair was performed. The conduit could be easily repositioned into the abdomen and the mesh was further tightened to the abdominal wall to prevent future events.

One patient developed an asymptomatic incisional hernia of the suprapubically placed Alexis port scar. The patient had been through many operations and was satisfied with the PSH repair, so she did not wish for further surgery, as long as she was asymptomatic.

One patient (4 previous PSH repairs; two direct, two Sugarbaker) developed a large hematoma in the previous PSH cavity, which was without pain or functional loss, so it was treated conservatively. Patient was discharged on postoperative day 5, but developed ischemia of the peristomal skin with blisters at day 6 and superficial necrosis at day 9. This was probably due to extensive adhesiolysis within the hernia cavity to achieve full mobilization of the stoma, leading to devascularization of the skin. As she was a referred patient, she was seen by her referring surgeon in her local hospital and treated conservatively in consultation with us. The ischemia subsided fully after 4 weeks. Full healing of the skin was achieved. A recurrence of the PSH developed after 4 months. A direct repair was performed, where we found the mesh had come loose from the abdominal wall. The mesh was refixed to the abdominal wall and to the conduit.

Two cases of clinically relevant seromas were seen, which were both treated conservatively.

## Discussion

The treatment of PSH is challenging, and more so in recurrent hernia, in cases where previously mesh has been placed.

**FIGURE 2:**
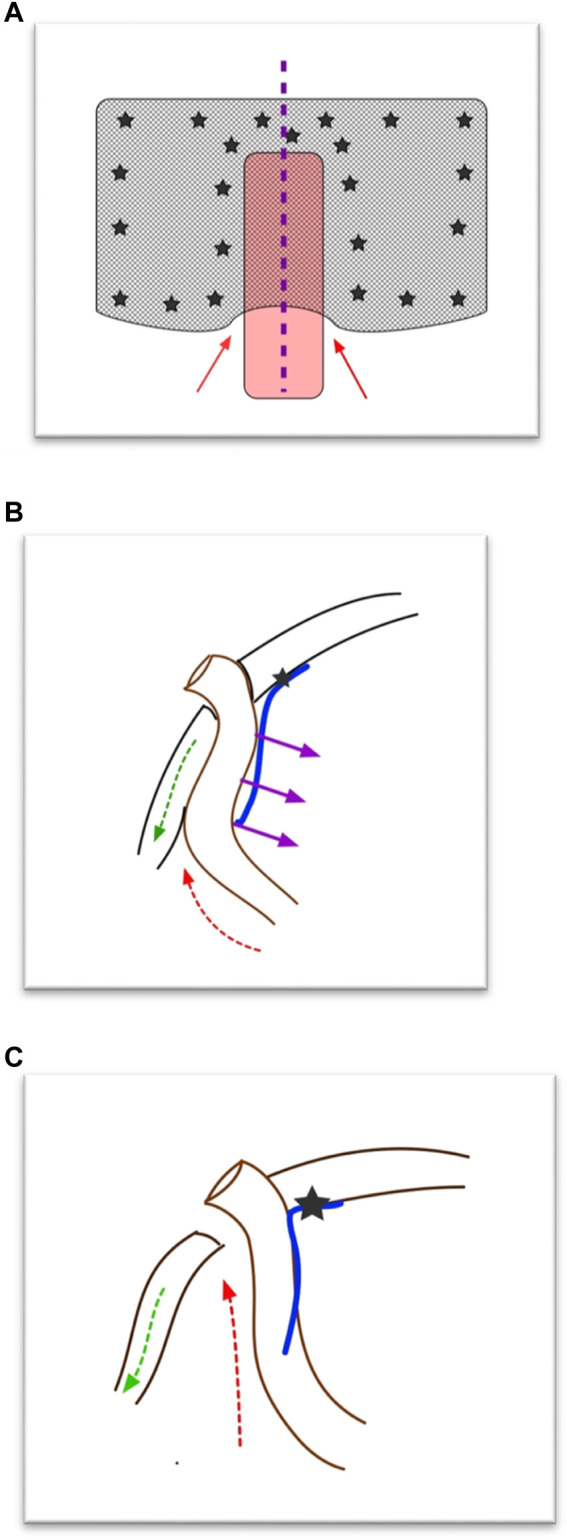
Hypothesis of development of parastomal hernia recurrence after (modified) Sugarbaker operation. **(A)** Front view of Sugarbaker repair. The red arrows depict the “weak spot.” The purple line depicts where in Panel **(B)** the cross section is shown. **(B)** Cross section of Sugarbaker repair. At the level of the conduit the mesh is only fixed medial to the stoma. The purple arrows depict the (counter)pressure of the bowel on the mesh. The green arrow depicts the retraction of the lateral abdominal muscles. The red arrow depicts the weak spot. **(C)** Cross section after Sugarbaker repair when the conduit has pressed away the mesh and the muscle has retracted. A recurrence will develop (red arrow).

The etiology of a recurrent PSH after a Sugarbaker (Figure 2) is unclear, but we believe that an important risk factor for recurrence of PSH is not placing the mesh with enough lateral overlap, which could, in time, lead to the development of space “behind” the conduit, as the conduit will slowly push the mesh of the peritoneum. Moreover, the abdominal wall directly lateral to the stomaconduit lacks connection to the “central tendon (linea alba),” which inevitably leads to retraction of the muscle at the level of the stoma, increasing the defect size. These two factors will result in a (cranio)lateral recurrence, which is the most often found recurrence after Sugarbaker in our experience. Another factor, we believe, that could lead to recurrence after IPOM Sugarbaker is the fact that we found that tackers almost never perforated the posterior fascia but were only in the peritoneum. The peritoneum is not strong enough to support the conduit and keep it attached to the lateral abdominal wall. Again, the conduit will push the mesh and the peritoneum from the abdominal wall and a recurrence can form laterally.

To this end, the placement of a mesh as deeply laterally as possible is of major importance.

We performed a robotic retromuscular repair of complex parastomal hernia using a synthetically reinforced biological mesh to durably treat even multiply recurrent PSH. Previous reports on robotic PSH repairs have shown comparable results, all in small numbers, to ours. Ayuso et al. describe 15 patients undergoing a robotic modified Sugarbaker PSH repair without any postoperative complications [16]. In this paper, however, no mention is made of the number of recurrent PSHs that were operated on. No previous placed meshes were described, which could explain a lower number of complications due to lower complexity of cases. An interesting adjustment to their Sugarbaker technique is the formation of a peritoneal flap lateral to the stoma to achieve deeper lateral placement of the mesh, which may be an important advantage of the Pauli repair compared to the “traditional” laparoscopic modified Sugarbaker technique.

Dewulf et al. report on their results for robotic Pauli PSH hernia repair in 26 patients, using a synthetic mesh [17]. In this series, 31% of the cases were recurrent hernias, with 4 meshes *in situ* and three cases had a prophylactic mesh *in situ*. Postoperative complications occurred in 8 out of 26 patients (31%), which is comparable to our results (3 out of 11; 27%). On the other hand, in our series no major complications, requiring reoperation, occurred. Dewulf et al. report on one case of stoma necrosis, necessitating revisional surgery and relocation of the stoma.

There are important limitations to this study. The numbers are small, and the follow-up is limited. The number of recurrences must therefore be taken with caution and longer follow-up is needed for reliable conclusions.

We hope this technique leads to a lower risk of mesh related complications such as erosion and infection, which are uncommon but devastating complications of synthetic mesh use in PSH repair [6–9], without compromising the endurance of the PSH-repair.

Despite the complexity of the operation and the large number of recurrent hernias with mesh *in situ*, the complications have been mild and curable in all cases.

## Data Availability

The original contributions presented in the study are included in the article/supplementary material, further inquiries can be directed to the corresponding author.
